# Light-Regulated Growth, Anatomical, Metabolites Biosynthesis and Transcriptional Changes in *Angelica sinensis*

**DOI:** 10.3390/plants13192744

**Published:** 2024-09-30

**Authors:** Hongyan Su, Xiuwen Cui, Yan Zhao, Mengfei Li, Jianhe Wei, Paul W. Paré

**Affiliations:** 1 State Key Laboratory of Arid Land Crop Science, College of Agronomy, Gansu Agricultural University, Lanzhou 730070, China; shy922322@163.com (H.S.); xiuwen2021@163.com (X.C.); 2 National and Local Joint Engineering Research Center on Germplasms Innovation and Utilization of Chinese Medicinal Materials in Southwest China, Yunnan Agricultural University, Kunming 650500, China; zhaoyankm@126.com; 3 Institute of Medicinal Plant Development, Chinese Academy of Medical Sciences & Peking Union Medical College, Beijing 100193, China; jhwei@implad.ac.cn; 4 Department of Chemistry and Biochemistry, Texas Tech University, Lubbock, TX 79409, USA; paul.pare@ttu.edu

**Keywords:** *Angelica sinensis*, white light, UV-B, bioactive metabolite, gene expression

## Abstract

*Angelica sinensis* is an alpine medicinal plant that has been widely used as a general blood tonic and gynecological indications over 2000 years, which depend on the bioactive metabolites (e.g., volatile oils, organic acids, and flavonoids). Although the accumulation of these metabolites is significantly affected by the environmental factors (e.g., altitude, temperature, and sunshine) as found in previous studies, the regulatory mechanism of different lights has not been clearly revealed. Here, growth parameters, contents of bioactive metabolites, and expression levels of related genes were examined when *A. sinensis* was exposed to different white-light (WL) and UV-B radiation treatments. The results showed that the differences in growth parameters (e.g., plant height, root length, and plant biomass) and leaf tissue characteristics (e.g., leaf thickness, stomatal density and shape, and chloroplast density) were observed under different light treatments. The contents of Z-ligustilide and ferulic acid elevated with the increase of WL (50 to 150 µmol·m^2^/s) and maximized under the combination of WL-100 and UV-B (107 µW/m^2^, UV-107) radiation, while the total flavonoids and polysaccharides contents, as well as in vitro antioxidant capacity, elevated with the increasing of WL and UV-B. mRNA transcripts encoding for the biosynthesis of volatile oils, ferulic acid, flavonoids, and polysaccharides were found to be differentially regulated under the different WL and UV-B treatments. These morphological, anatomical, and transcriptional changes are consistent with the elevated bioactive metabolites in *A. sinensis* under the combination of WL and UV-B. These findings will provide useful references for improving bioactive metabolite production via the cultivation and bioengineering of *A. sinensis*.

## 1. Introduction

*Angelica sinensis* (Oliv.) Diels (family Apiaceae) is a perennial rhizomatous herb that is originally native to northwest China, thriving in wet and cold regions at altitudes of 2200 to 3000 m [1,2]. The roots (renowned as dang gui) have been widely used as a general blood tonic and for gynecological indications over 2000 years, and for the treatment of cardiovascular, hepatoprotective, and gynecological diseases in recent years [1]. The most important constituents associated with the biological activity are thought to be alkylphthalides (e.g., Z-ligustilide) and possibly ferulic acid, flavonoids, and polysaccharides [1,3,4]. 

Z-ligustilide and ferulic acid are often used as the quality marker (Q-mark) for the assessment of dang gui [1,5]. To date, the biosynthetic pathway of phthalides belonging to volatile oils, especially in Z-ligustilide, has not been mapped [6,7,8], while several genes participating in the biosynthesis of volatile oils have been previously identified in RNA-seq of *A. sinensis* [9,10,11], such as the *t-anol/isoeugenol O-methyltransferase 1* (*AIMT1*) involved in converting trans-anol to trans-anethole and isoeugenol to isomethyleugenol [12], *acetyl-CoA-benzylalcohol acetyltransferase* (*BEAT*) involved in the biosynthesis of benzyl acetate [13], and *cytochrome P450 705A1* (*CYP705A1*) involved in the biosynthesis of the volatile homoterpene (E)-4,8-dimethyl-1,3,7-nonatriene [14]. Presently, the biosynthetic pathway of ferulic acid and flavonoids belonging to the phenylpropanoids has been mapped in plants, and the genes identified from *A. sinensis* and related to ferulic acid biosynthesis include *phenylalanine ammonia lyase* (*PAL*), *cinnamate 4-hydroxylase* (*C4H*), *p-coumarate 3 hydroxylase* (*C3H*), and *caffeic acid 3-O-methyltransferase* (*COMT*), as well as to flavonoids biosynthesis include: *chalcone synthase* (*CHS*), *chalcone isomerase* (*CHI*), and *UDP-glycosyltransferases* (*UGTs*), amongst others (Figure S1) [9,10,11,15,16]. 

Geographical modeling on *A. sinensis* has predicted that the four environmental factors (i.e., altitude, temperature, sunshine, and rainfall) play determining roles in plant growth and biosynthesis of metabolites (e.g., Z-ligustilide, ferulic acid, and butenyl phthalide) [7,17]. Extensive experiments have demonstrated that there is a significant positive correlation between the content of metabolites (i.e., Z-ligustilide, ferulic acid, butenyl phthalide, flavonoids, polysaccharides, and phenolics) and the increase in altitude from 2000 to 2900 m [18,19,20,21,22,23].

Generally, there is a decrease in temperature and an increase in sunshine and rainfall with increasing altitude [24]. Previous studies on *A. sinensis* have found that a cooler temperature (15 °C) improves plant biomass, the contents of ferulic acid and total flavonoids, and expression levels of related genes (e.g., *PAL*, *C3H*, and *CHS*), while decreasing polysaccharides content compared with 22 °C [15]; soil drought decreases root biomass and the content of ferulic acid and volatile oils [25]; reducing light intensity with 50% to 75% sunshade improves root biomass and ferulic acid content [26,27], and an increase of UV-B radiation improves phthalide content [28]. 

Extensive experiments have demonstrated that light, especially in UV-B, significantly affects plant growth and regulates metabolites biosynthesis, such as chlorogenic acid in *Scutellaria baicalensis* [29], flavonoids in *Chrysanthemum morifolium* [30], and lignans in *Schisandra chinensis* [31]. Although previous studies have indicated that there were significant effects of light intensities and UV-B radiation on plant growth (i.e., root biomass) and the content of some metabolites (i.e., ferulic acid and phthalides) in *A. sinensis* [26,27,28], the regulatory mechanism of growth characteristics, metabolites accumulation, and related genes expression has not been examined when plants are exposed to synergistic white-light (WL) and UV-B radiation. In the present study, we probe the role of leaf tissue structures, metabolites content, and expression level of genes related to the biosynthesis of volatile oils, ferulic acid, flavonoids, and polysaccharides to identify possible links between light and metabolites accumulation.

## 2. Results

### 2.1. Changes in Growth Parameters in Response to White-Light (WL) and UV-B Radiation

In this study, significant differences in plant growth parameters (i.e., plant height, root length, aerial parts and root dry weight, and chlorophyll content) were observed under different light treatments. Specifically, there was a significant decrease in plant height, root length, aerial parts and root dry weight, and chlorophyll (a + b) content under the 50 µmol·m^2^/s white-light (WL-50), 100 µmol·m^2^/s white-light [WL-100, control (CK)] supplemented with a 107 µW/m^2^ UV-B (CK+UV-107), and CK supplemented with a 214 µW/m^2^ UV-B (CK+UV-214), while a 1.03-, 1.06-, 1.08-, 1.04-, and 1.05-fold increase was observed under the 150 µmol·m^2^/s white-light (WL-150) compared with CK, respectively. These results indicated that the WL-150 was more beneficial to plant growth (Table 1 and Figure S2).

### 2.2. Changes in Cell Micro-Structure and Leaf Stomata in Response to WL and UV-B Radiation

**Figure 1:**
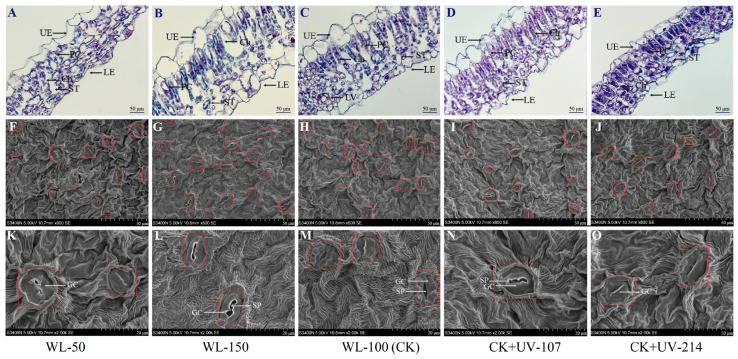
Changes in cell micro- and leaf stomata structure of *A. sinensis* in response to white-light (WL) and UV-B. WL-100 as a reference (CK); Ch: chloroplast; GC: guard cell; LE: lower epidermis; PC: palisade cell; SP: stomatal pore; ST: spongy tissue; UE: upper epidermis. Images (**A**,**F**,**K**) represent the cell micro- and leaf stomata structure under WL-50 treatment; images (**B**,**G**,**L**) represent the structure under WL-150 treatment; images (**C**,**H**,**M**) represent the structure under WL-100 (CK) treatment; images (**D**,**I**,**N**) represent the structure under CK + UV-107 treatment; and images (**E**,**J**,**O**) represent the structure under CK + UV-214 treatment.

Significant alteration of leaf tissue structure (e.g., leaf thickness, chloroplast number, and stomata characteristics) was observed under different light treatments (Figure 1). Specifically, an increase in leaf thickness and chloroplast number was observed with the increase of WL from 50 to 150 µmol·m^2^/s (Figure 1A–C), while a decrease was observed with the increase of UV-B radiation from 0 to 214 µW/m^2^ (Figure 1C–E). Similarly, there were 1.24- and 1.40-fold as well as 1.97- and 2.22-fold increases in stomata density and stomatal opening percentage under the CK and WL-150 compared with WL-50 (Figure 1F–H,K–M), while there were 1.38- and 1.57-fold as well as 1.36- and 2.32-fold decreases under the CK+UV-107 and CK+UV-214 compared with CK (Figure 1H–J,M–O; Table S1). The greater stomatal opening percentage under the CK and WL-150 will contribute to an increase in the net CO_2_ fixation rate during photosynthesis and overall biomass accumulation. These results further indicated that plant growth could be improved under the WL compared with UV-B radiation. 

### 2.3. Changes in Cell Ultra-Structure in Response to WL and UV-B Radiation

**Figure 2:**
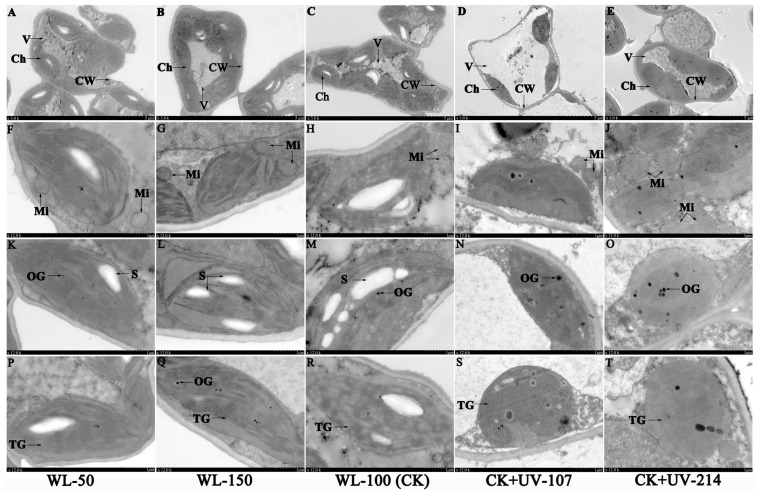
Changes in cell ultra-structure of *A. sinensis* leaf in response to WL and UV-B. WL-100 as a reference (CK); CW: cell wall; Ch: chloroplast; Mi: mitochondria; S: starch grain; TG: thylakoid grana; OG: osmiophilic granule; V: vacuole. The scale in the first line shows 5 µm, and the scales in second to fourth lines show 1 µm. Images (**A**,**F**,**K**,**P**) represent the cell ultra- structure under WL-50 treatment; images (**B**,**G**,**L**,**Q**) represent the structure under WL-150 treatment; images (**C**,**H**,**M**,**R**) represent the structure under WL-100 (CK) treatment; images (**D**,**I**,**N**,**S**) represent the structure under CK + UV-107 treatment; and images (**E**,**J**,**O**,**T**) represent the structure under CK + UV-214 treatment.

The leaf cell ultra-structures were significantly affected by different light treatments (Figure 2). Specifically, vacuole (V) occupied most of the space of the whole cell, and chloroplasts (Chs) were near to the cell wall (CW) (Figure 2A–E); mitochondria (Mi) were near the Ch (Figure 2F–J); starch grains (Ss) (Figure 2K–M), osmiophilic granules (OGs) (Figure 2K,M–O,Q), and thylakoid grana (TG) (Figure 2P–T) were present in the Ch. The number of Ch, S, TG, and OG obviously appeared to be greater under the CK and WL-150 compared with WL-50, while the number of Ch appeared to be lower under the CK+UV-107 and CK+UV-214 compared with CK (Figure 2A–E,K–T); the number of S gradually increased with the increase of WL, while it completely disappeared under UV-B radiation (Figure 2K–O). These changes in cell ultra-structure are consistent with the plant parameters under different light treatments.

### 2.4. Changes in Contents of Z-Ligustilide and Ferulic Acid in Response to WL and UV-B Radiation

**Figure 3:**
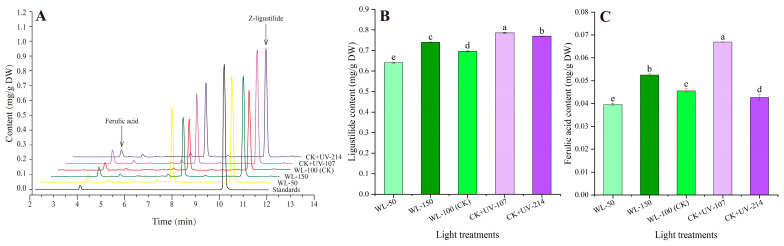
Changes in the contents of Z-ligustilide and ferulic acid, as well as their representative chromatogram, in response to WL and UV-B. WL-100 is usedas a reference (CK); Image (**A**) shows the representative chromatogram of the ferulic acid and Z-ligustilide; Image (**B**) shows the content of Z-ligustilide; and Image (**C**) shows the content of ferulic acid. Different lowercase letters represent significant differences (*p* < 0.05) among different treatments.

As shown in Figure 3, the representative chromatogram of Z-ligustilide and ferulic acid is shown in Figure 3A, and significant differences in Z-ligustilide and ferulic acid were observed under different light treatments. Specifically, a 1.06-, 1.13-, and 1.11-fold increase in Z-ligustilide content was observed under the WL-150, CK+UV-107, and CK+UV-214, while a 1.09-fold decrease was observed under the WL-50 compared with CK, respectively (Figure 3B); a 1.15- and 1.47-fold increase in ferulic acid content was observed under the WL-150 and CK+UV-107, while a 1.15- and 1.07-fold decrease was observed under the WL-50 and CK+UV-214 compared with CK, respectively (Figure 3C). 

### 2.5. Changes in Contents of Bioactive Metabolites and Antioxidant Capacity in Response to WL and UV-B Radiation

**Figure 4:**
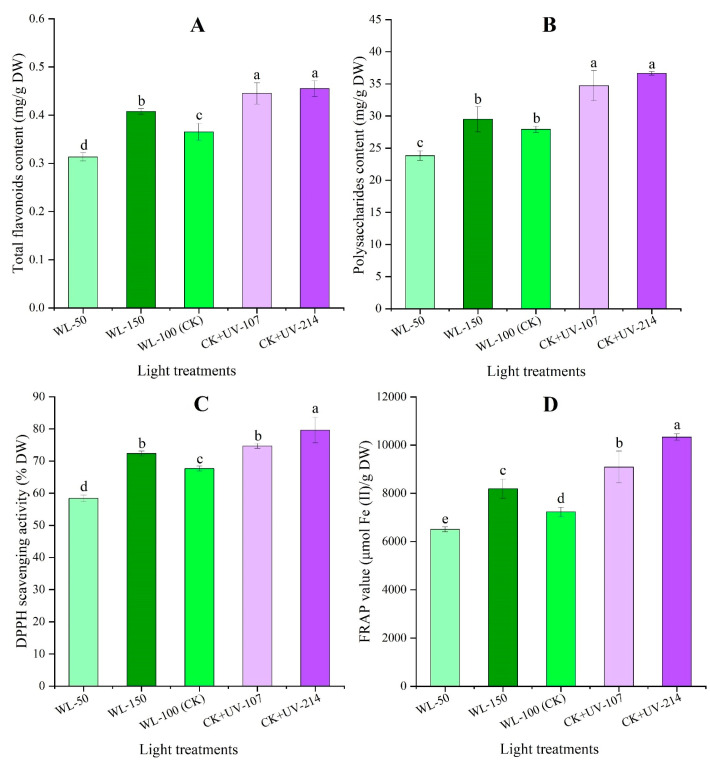
Changes in the contents of total flavonoids (**A**), polysaccharides (**B**), DPPH scavenging activity (**C**), and FRAP value (**D**) in rhizomes of *A. sinensis* in response to WL and UV-B. WL-100 as a reference (CK); different lowercase letters represent significant differences (*p* < 0.05) among different treatments.

Significant differences in bioactive metabolites (total flavonoids and polysaccharides) and antioxidant capacity [1,1-diphenyl-2-prcrylhydrazyl (DPPH) and Ferric reducing/antioxidant power (FRAP)] were observed under different light treatments (Figure 4). Specifically, a 1.12-, 1.22-, and 1.25-fold as well as 1.06-, 1.24, and 1.31-fold increase in total flavonoids and polysaccharides contents were observed under the WL-150, CK+UV-107, and CK+UV-214, while a 1.17- and 1.17-fold decrease was observed under the WL-50 compared with CK (Figure 4A,B). Similarly, a 1.07-, 1.10-, and 1.18-fold as well as 1.13-, 1.26-, and 1.43-fold increase of DPPH scavenging activity and FRAP value were observed under the WL-150, CK+UV-107, and CK+UV-214, while a 1.16- and 1.11-fold decrease was observed under the WL-50 compared with CK (Figure 4C,D). These results showed that higher WL and UV-B radiation could improve the accumulation of total flavonoids and polysaccharides in *A. sinensis*.

### 2.6. Changes in Gene Expression in Response to WL and UV-B Radiation

#### 2.6.1. Changes in Expression Level of Genes Related to Volatile Oil Biosynthesis

**Figure 5:**
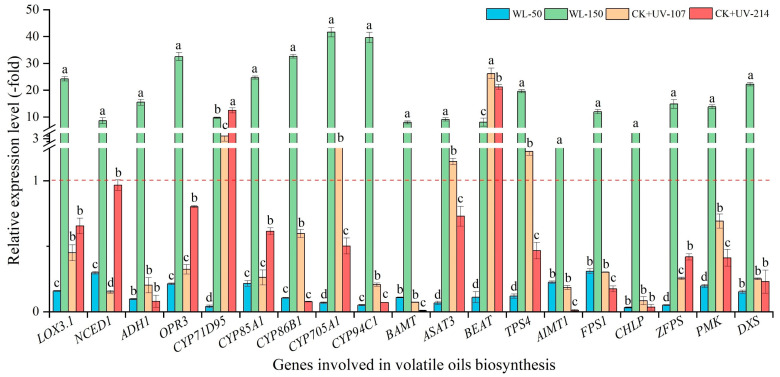
Expression levels of genes related to volatile oils biosynthesis in response to WL and UV-B. WL-100 as a reference (CK); different lowercase letters represent significant differences (*p* < 0.05) among different treatments.

As shown in Figure 5, the mRNA expression level of the selected 19 genes related to volatile oil biosynthesis showed a significant alteration under different light treatments, with a 1.56- (*AIMT1*) to 41.64-fold (*CYP705A1*) up-regulation (UR) under WL-150, while a 0.03- (*CHLP*) to 0.31-fold (*FPS1*) down-regulation (DR) under WL-50 compared with CK; a 1.15- (*ASAT3*) to 26.22-fold (*BEAT*) UR for the five genes (i.e., *CYP71D95*, *CYP705A1*, *ASAT3*, *BEAT*, and *TPS4*) and a 12.49- (*CYP71D95*) to 21.22-fold (*BEAT*) UR, while a 0.08- (*CHLP*) to 0.69 -fold (*PMK*) DR and 0.01- (*BAMT*) to 0.97-fold (*NCED1*) DR under CK+UV-107 and CK+UV-214 compared with CK, respectively.

#### 2.6.2. Changes in Expression Level of Genes Related to Ferulic Acid Biosynthesis

**Figure 6:**
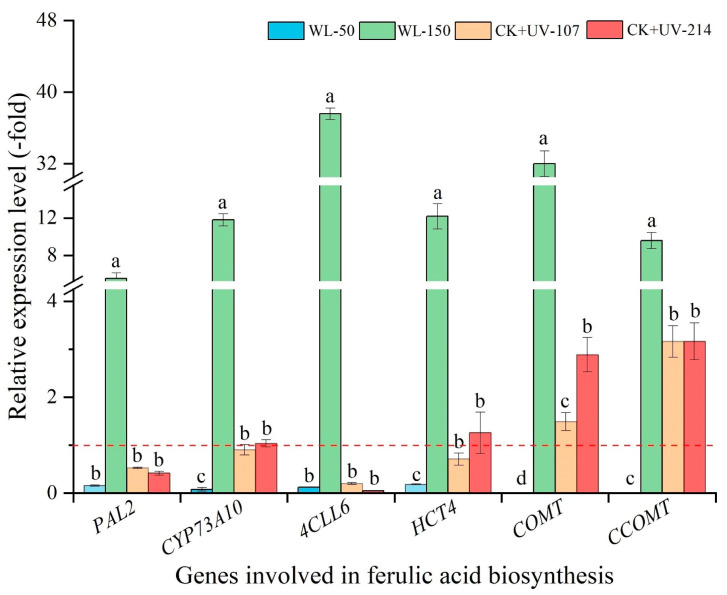
Expression levels of genes related to ferulic acid biosynthesis in response to WL and UV-B. WL-100 as a reference (CK); different lowercase letters represent significant differences (*p* < 0.05) among different treatments.

As shown in Figure 6, the mRNA expression level of the selected six genes related to ferulic acid biosynthesis showed a significant alteration under different light treatments, with a 5.54- (*PAL2*) to 37.58-fold (*4CLL6*) UR under WL-150, while a 0.0007- (*COMT* and *CCOMT*) to 0.18-fold (*HCT4*) DR under WL-50 compared with CK; with a 1.50- (*COMT*) to 3.16-fold (*CCOMT*) UR and a 1.04- (*CYP73A10*) to 3.16-fold (*CCOMT*) UR, while a 0.53- (*PAL2*) to 0.90-fold (*CYP73A10*) DR and a 0.42- (*PAL2*) to 0.05-fold (*4CLL6*) DR under CK+UV-107 and CK+UV-214 compared with CK, respectively. The change trend of the expression level of the six key genes is almost consistent with the content of ferulic acid (Figure 3C and Figure 6). 

#### 2.6.3. Changes in Expression Level of Genes Related to Flavonoids Biosynthesis

**Figure 7:**
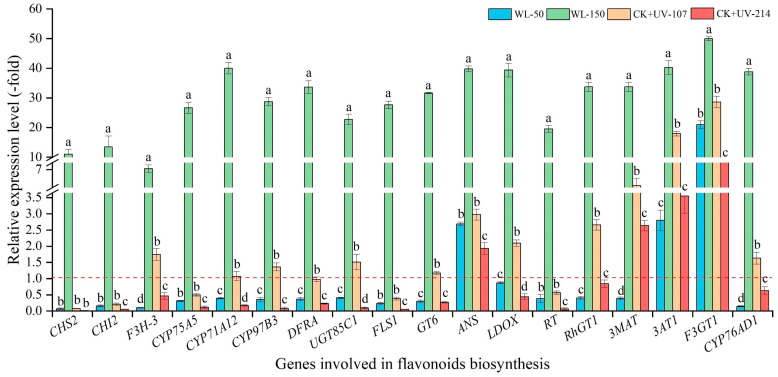
Expression levels of genes related to flavonoids biosynthesis in response to WL and UV-B. WL-100 as a reference (CK); different lowercase letters represent significant differences (*p* < 0.05) among different treatments.

As shown in Figure 7, the mRNA expression level of the selected 18 genes related to flavonoid biosynthesis showed a significant alteration under different light treatments, with a 7.03- (*F3H-3*) to 49.92-fold (*F3GT1*) UR under WL-150 compared with CK; with a 2.69- (*ANS*) to 20.95-fold (*F3GT1*) UR, 1.07- (*CYP71A12*) to 28.63-fold (*F3GT1*) UR, and 1.93- (*ANS*) to 8.52-fold (*F3GT1*) UR, while a 0.07- (*CHS2*) to 0.87-fold (*LDOX*) DR, 0.07- (*CHS2*) to 0.98-fold (*DFRA*) DR, and 0.01- (*CHS2*) to 0.84-fold (*RhGT1*) DR under WL-50, CK+UV-107, and CK+UV-214 compared with CK, respectively. The change trend of the expression level of the 18 key genes is almost consistent with the content of flavonoids (Figure 4A and Figure 7).

#### 2.6.4. Changes in Expression Level of Genes Related to Polysaccharides Biosynthesis

**Figure 8:**
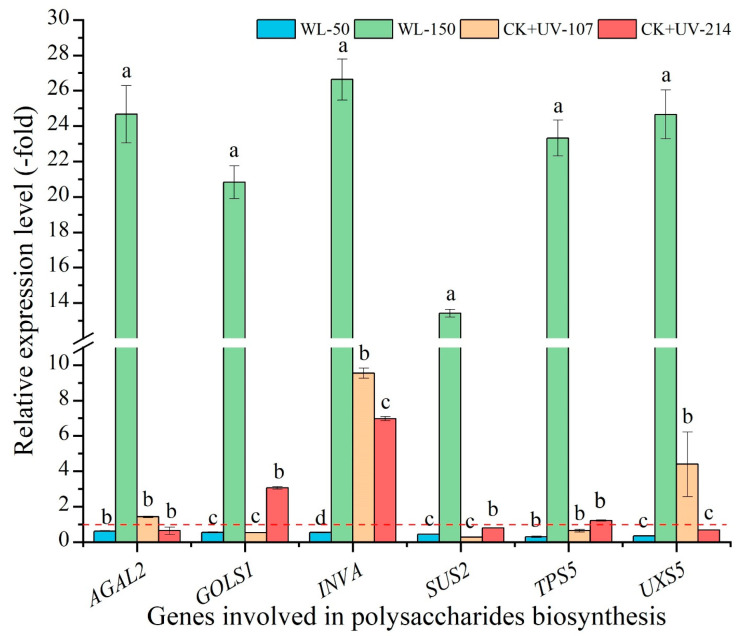
Expression levels of genes related to polysaccharides biosynthesis in response to WL and UV-B. WL-100 as a reference (CK); different lowercase letters represent significant differences (*p* < 0.05) among different treatments.

As shown in Figure 8, the mRNA expression level of the selected six genes related to polysaccharides biosynthesis showed a significant alteration under different light treatments, with a 13.42- (*SUS2*) to 63.33-fold (*TPS5*) UR under WL-150 compared with CK, while a 0.29- (*TPS5*) to 0.62-fold (*AGAL2*) DR under WL-50 compared with CK; with a 1.43- (*AGAL2*) to 9.54-fold (*INVA*) UR and 1.22- (*TPS5*) to 6.97-fold (*INVA*) UR, while a 0.27- (*SUS2*) to 0.64-fold (*TPS5*) DR and 0.68- (*UXS5*) to 0.80-fold (*SUS2*) DR under CK+UV-107 and CK+UV-214 compared with CK, respectively. The change trend of the expression level of the six key genes is almost consistent with the content of polysaccharides (Figure 4B and Figure 8).

## 3. Discussion

Light plays critical roles in affecting plant growth and metabolite biosynthesis, with different plants responding to different light conditions [32,33]. In this study, the growth and leaf tissue characteristics, contents of bioactive metabolites, and expression levels of related genes were significantly affected when *A. sinensis* was exposed to white-light (WL) and UV-B radiation.

Previous studies have found that the plant growth improved by appropriate light, whereas inhibition by UV-B radiation has been observed in other plants. For example, there was an increase of plant growth (e.g., plant height, leaf number and area, and biomass) and photosynthetic ability of *Sinopodophyllum hexandrum* and *Mahonia bodinieri* under moderate light [34,35]; while there was a decrease in plant growth (e.g., plant height, fresh weight of leaves, shoots and roots as well as leaf area) of *Avena fatua* and *Setaria viridis* under different doses of UV-B radiation [36]. For *A. sinensis*, root biomass and ferulic acid content respectively showed a 1.13- and 1.18-fold increase, respectively, under 75% sunshade compared with 50% sunshade [26,27]; and the phthalide content showed a 1.29-fold increase under UV-B radiation compared with CK [28]. In this study, significant differences in plant growth were observed under different light treatments (Table 1 and Figure S2).

For the leaf tissue structure, previous studies on other plants have found that the leaf tissue structure is significantly affected by light intensity and quality; for example, there was an increase in leaf thickness (e.g., palisade parenchyma, spongy parenchyma, and lower epidermis) of *Petunia* × *hybrida* and stomatal density of *M. bodinieri* with increasing light intensity [34,37], while a decrease in leaf thickness occurred(e.g., epidermis, palisade, and mesophyll) with increasing UV-B radiation [38], as well as a decrease in stomatal opening rate of *Cucumis sativus* under UV-B radiation [39]. In this study, significant alterations of leaf tissue structure (e.g., leaf thickness, chloroplast number, and stomata characteristics) were observed under different light treatments (Figure 1). Previous studies on other plants have found that leaf ultra-structure is also significantly affected by light intensity and quality; for example, an increase in Ch and S numbers in *Lycopersicon esculentum* was observed with the increasing light intensity [40]; the Ch became coarsened and shortened, TG was swollen and distorted, and the number of OGs increased in Morus alba under increasing UV-B radiation [41]. In this study, significant alterations of leaf ultra-structure (e.g., Ch, TG, and OG) were also observed under different light treatments (Figure 2). These changes were consistent with the changes in chlorophyll (a + b) content, and the increase of photosynthetic components (i.e., Ch and TG) plays critical roles in energy acquisition and metabolites storage (i.e., S) for greater biomass [42].

For the bioactive metabolites, previous studies on *A. sinensis* have found that the Z-ligustilide content showed a 1.28-fold increase under UV-B radiation compared with CK [28]; the ferulic acid content showed a 1.18-fold increase under 75% sunshade compared with 50% sunshade [27]; and the Z-ligustilide and ferulic acid contents showed a 1.05- and 1.14-fold increase at 2780 m compared with 2360 m [21]. In this study, significant alteration of Z-ligustilide and ferulic acid content was observed under different light treatments (Figure 3). Extensive experiments have demonstrated that there is a significant positive correlation between total flavonoids and polysaccharides contents with in vitro antioxidant capacity in plants [43,44,45]. Previous studies on other plants have found that the total flavonoids and polysaccharides contents and in vitro antioxidant capacity were enhanced with an appropriate dose of light intensity and UV-B radiation [45,46,47]. In this study, the total flavonoids and polysaccharides contents, as well as in vitro antioxidant capacity, elevated with increasing WL and UV-B (Figure 4).

For the volatile oil biosynthesis, previous studies have found that genes (e.g., *LOX3.1*, *NCED1*, and *ADH1*) participate in the biosynthesis of volatile oils [9,10,11,15]. For example, the *BAMT* is involved in converting benzoic acid into the volatile ester methyl benzoates [48]; the *LOX3.1* is involved in catalyzing the hydroperoxidation of lipids containing a pentadiene structure [49]; and the *ZFPS* is involved in the biosynthesis of several sesquiterpenes [50]. For the ferulic acid biosynthesis, previous studies have demonstrated that the genes (e.g., *PAL2*, *CYP73A10*, and *4CLL6*) are directly participating in ferulic acid biosynthesis (Figure S1) [8,9,10,15,16]. In this study, these genes’ expression levels were almost consistent with the ferulic acid content (Figure 3C and Figure 6).

Extensive studies have demonstrated that flavonoids exhibit multi-biological functions in plants [51,52]. Previous studies have found that some genes participate in flavonoid biosynthesis (Figure S1) [15]. For example, the *CYPs* are involved in flavonoid and pigment biosynthesis [53,54]; and the *GT6* is involved in the biosynthesis of flavonol 3-O-glucosides [55]. These genes’ expression levels were almost consistent with the flavonoids content (Figure 4A and Figure 7). For polysaccharides biosynthesis, previous studies have found that these genes participate in polysaccharides biosynthesis [11]. For example, the *GOLS1* is involved in the biosynthesis of raffinose family oligosaccharides [56]; the *INVA* is involved in converting sucrose into glucose and fructose [57]; and the *TPS5* is involved in the starch and sucrose degradation [58]. These genes’ expression levels were almost consistent with the polysaccharides content (Figure 4B and Figure 8).

**Figure 9:**
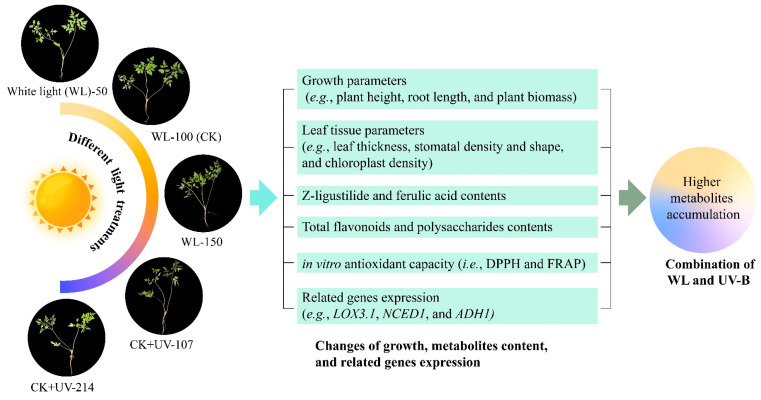
A proposed model of light-regulated growth, anatomical changes, metabolite biosynthesis, and transcriptional changes in *A. sinensis*.

Based on the results above, a model of light-regulated growth, anatomical, metabolites biosynthesis, and transcriptional changes in *A. sinensis* is proposed (Figure 9). Specifically, when plants are exposed to different lights, there are significant alterations in growth and leaf tissue parameters, bioactive metabolites accumulation, and in vitro antioxidant capacity. The changes in related gene expression (e.g., *LOX3.1*, *NCED1*, and *ADH1*) are almost consistent with the growth parameters (e.g., plant height, root length, and plant biomass), leaf tissue parameters (e.g., leaf thickness, stomatal density and shape, and chloroplast density), and contents of bioactive metabolites (i.e., Z-ligustilide, ferulic acid, total flavonoids, and polysaccharides). These findings demonstrate that the combination of WL and UV-B treatments can promote plant growth and metabolites accumulation in *A. sinensis*.

## 4. Materials and Methods

### 4.1. Plant Materials

The seedlings (root shoulder diameter 0.4–0.5 cm) developed from the mature seeds of 3-year-old plants of *Angelica sinensis* (Oliv.) Diels (cultivar Mingui 1) were stored in a refrigerator at 0 °C for 50 days [59]. The species was identified by Professor Mengfei Li (Gansu Agricultural University, Lanzhou, Gansu, China). Then, the stored seedlings were transplanted to pots (17 cm × 20 cm; two seedlings per pot) with a nutrition matrix (peat moss, organic content > 98%; soil relative water content 60–70%) and grown under a 12/12 h light/dark photocycle (white light 100 µmol/m^2^/s photon flux density) at 20 °C and 60% air relative humidity in a greenhouse. 

After 30 days of growth, the plants were divided into 5 groups. Specifically, the first group continued growing under the WL-100 (CK); the second group was transferred to WL-50; the third group was transferred to WL-150; the fourth group was transferred to CK+UV-107 at 306 nm for 3 h per day (G40T10E, SANKYO DENKI, Kanagawa, Japan); and the fifth group was transferred to CK+UV-214 at 306 nm for 3 h per day. Each group has 20 repeats (20 pots × 2 seedlings per pot = 40 seedlings). 

After 15 days of growth under 5 treatments, the plants were harvested, and the plant height and root length were measured (*n* = 40). Thereafter, 10 fresh plants were used for the measurement of chlorophyll content and leaf tissue structures; 10 fresh plants were stored at −80 °C for the determination of related gene expression; the other 20 plants were dried at room temperature for the determination of dry weight (DW) of aerial parts and roots, main metabolite content, and in vitro antioxidant capacity.

### 4.2. Measurement of Chlorophyll Content, Leaf Stomata, and Cell Structure

The total chlorophyll content was measured using a spectrophotometer (UV-6100, Shanghai, China) at 665 and 649 nm according to previous protocols [60]; leaf stomata were measured using a scanning electron microscope (SEM, S-3400N, Hitachi, Tokyo, Japan) according to previous protocols [61]; cell micro-structure was measured using an inverted microscope (Revolve RVL-100-G, ECHO, Lake Zurich, IL, USA), and cell ultra-structure was measured using a transmission electron microscope (TEM, JEM1230, JEOL Ltd., Tokyo, Japan) according to previous protocols [62]. 

### 4.3. Determination of Bioactive Metabolites Content and Antioxidant Capacity

The fine powder (1.5 g) of air-dried roots was soaked in 95% ethanol (20 mL) and agitated at 25 °C and 120 r/min for 48 h. The mixture was centrifuged at 4 °C and 5000 r/min for 10 min. The extracts were increased to 20 mL with 95% ethanol for the determination of bioactive metabolites (Z-ligustilide, ferulic acid, total flavonoids, and polysaccharides) contents and in vitro antioxidant capacities (DPPH scavenging activity and FRAP value).

The content of Z-ligustilide and ferulic acid was determined using a high-performance liquid chromatography (HPLC) according to previous protocols [63]. The content of total flavonoids, polysaccharides, and in vitro antioxidant capacity (DPPH and FRAP) was determined using the spectrometer (UV-6100, Shanghai, China) at 510 nm, 485 nm, 515 nm, and 593 nm, respectively. Specifically, the total flavonoids content used a NaNO_2_-AlCl_3_-NaOH method [64]; the polysaccharides content used the phenolsulfuric acid method [65]; the DPPH scavenging activity used a scavenging of DPPH radicals [66]; and the FRAP value used a reduction of ferric-tripyridyltriazine complex [67]. The roots from the 20 dried plants were mixed, and all the determinations have 3 technical repeats.

### 4.4. Quantification of Gene Expression

A total of 49 genes was selected based on the RNA-seq of *A. sinensis* in our previously published literature [9,10,11], including: 19 genes related to volatile oils biosynthesis, 6 genes related to ferulic acid biosynthesis, 18 genes related to flavonoids biosynthesis, and 6 genes related to polysaccharides biosynthesis. Their primer sequences (Table S2) were designed using a tool of primer-blast in the National Center for Biotechnology Information (NCBI). Total RNA was extracted from the roots using an RNA kit, and the purity was determined using a NanoDrop. First-strand cDNA was synthesized using a FastKing RT kit, and PCR amplification was performed using a SuperReal PreMix. Gene expression was quantified by qRT-PCR. *Actin* (*ACT*) gene [8] was used as an internal reference, and relative expression level (REL) of the gene was calculated using a 2^−ΔΔ^*^Ct^* method [68]. The roots from the 10 freezing fresh plants were mixed, and all the determinations have 3 technical repeats.

### 4.5. Statistical Analysis

Statistical analysis was performed using Duncan’s multiple range test in SPSS 22.0 with *p* < 0.05 considered as a significant difference.

## 5. Conclusions

Based on the above observations, light is an important environmental factor that regulates growth and bioactive metabolites biosynthesis, as well as related genes’ expression levels in *A. sinensis*, with the combination of WL and UV-B radiation altering growth characteristics (e.g., plant height, root length, and plant biomass) and leaf tissue structures (e.g., leaf thickness, stomatal density and shape, and chloroplast density), and enhancing the accumulation of bioactive metabolites (e.g., Z-ligustilide, ferulic acid, and flavonoids) via the differential expression of related genes. These findings will provide useful references for improving bioactive metabolites production via the cultivation and bioengineering of *A. sinensis*.

## Figures and Tables

**Table 1:** Changes in growth parameters of *A. sinensis* in response to white-light (WL) and UV-B.

Growth Parameters	WL-50	WL-150	WL-100 (CK)	CK+UV-107	CK+UV-214
Plant height (cm)	20.34 ± 0.83 ^d^	23.34 ± 1.69 ^a^	22.62 ± 1.31 ^b^	21.46 ± 1.29 ^c^	20.13 ± 0.79 ^d^
Root length (cm)	10.81 ± 0.55 ^c^	12.08 ± 0.73 ^a^	11.42 ± 0.88 ^b^	11.30 ± 0.65 ^b^	10.45 ± 0.45 ^d^
Aerial parts dry weight (g)	0.22 ± 0.02 ^c^	0.26 ± 0.02 ^a^	0.24 ± 0.02 ^b^	0.22 ± 0.02 ^c^	0.20 ± 0.02 ^d^
Root dry weight (g)	0.20 ± 0.02 ^c^	0.24 ± 0.03 ^a^	0.23 ± 0.03 ^a^	0.21 ± 0.02 ^b^	0.19 ± 0.02 ^c^
Chlorophyll (a + b) (mg/g FW)	1.54 ± 0.03 ^c^	1.77 ± 0.02 ^a^	1.69 ± 0.01 ^b^	1.38 ± 0.03 ^d^	1.04 ± 0.02 ^e^

Note: Different letters in the same line show a significant difference at *p* < 0.05 level among different treatments.

## Data Availability

The datasets of transcriptomics are publicly available at NCBI, with accession: PRJNA591308 and ID: 591308 (https://www.ncbi.nlm.nih.gov/bioproject/PRJNA591308, accessed on 20 August 2021), accession SAMN24046640 to SAMN24046648; SRA accession SRR17235563 to SRR17235571 (https://www.ncbi.nlm.nih.gov/bioproject/PRJNA789039, accessed on 14 December 2021), and Sequence Read Archive (SRA) accession: SRR16993328 to SRR16993332.

## Supplementary Materials

The following supporting information can be downloaded at: https://www.mdpi.com/article/10.3390/plants13192744/s1, Figure S1: Schematic representation of biosynthetic pathways leading from shikimic acid pathway to phenylpropanoid pathway. Solid arrow indicates known steps, whereas multiple arrows indicate multiple reaction steps. Enzyme abbreviations are as follows: EMB3004, Bifunctional 3-dehydroquinate dehydratase/shikimate dehydrogenase, chloroplastic; CM, chorismate mutase; PAL, phenylalanine ammonia lyase; C4H, cinnamate 4-hydroxylase; 4CL, 4-coumarate-CoA ligase; HCT, hydroxycinnamoyl shikimate transferase; C3H, p-coumarate 3-hydroxylase; CCOAMT, caffeoyl-CoA 3-O-methyltransferase; CCR, cinnamoyl CoA oxidoreductases; COMT, caffeic acid 3-O-methyltransferase; CHS, chalcone synthase; CHI, chalcone isomerase. ① Showing the ferulic acid biosynthesis via CCOAMT sub-pathway; ② Showing the ferulic acid biosynthesis via COMT sub-pathway;③ Showing the flavonoid biosynthetic sub-pathway (15); Figure S2: Morphological characteristics of *A. sinensis* in response to WL and UV-B. Table S1: Stomata parameters of *A. sinensis* in response to WL and UV-B (mean ± SD, *n* = 10); Table S2: Primer sequences used to amplify the 49 genes related to the biosynthesis of volatile oils, ferulic acid, flavonoids, and polysaccharides.
