# Supplementary material for: Light-Regulated Growth, Anatomical, Metabolites Biosynthesis and Transcriptional Changes in Angelica sinensis
Source: Plants (Basel). 2024 Sep 30;13(19):2744. doi: 10.3390/plants13192744 (PMC11478417; doi:10.3390/plants13192744)
Supplement: Supplementary file 1 [file plants-13-02744-s001.zip › plants-3160827-supplementary.pdf]

Supplemental Materials

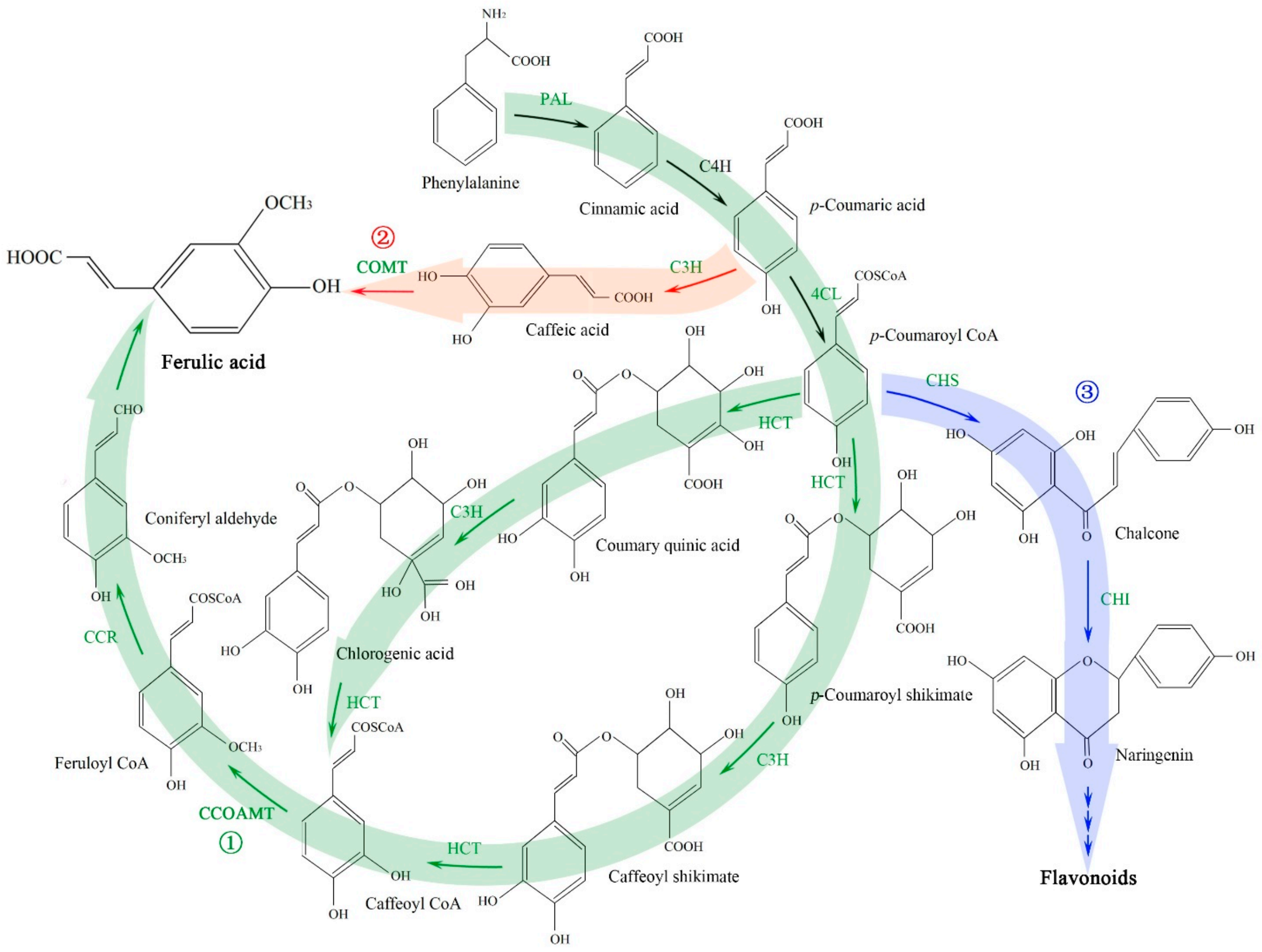

Figure S1. Schematic representation of biosynthetic pathways leading from shikimic acid pathway to phenylpropanoid pathway. Solid arrow indicates known steps, whereas multiple arrows indicate multiple reaction steps. Enzyme abbreviations are as follows: EMB3004, Bifunctional 3-dehydroquinate dehydratase/shikimate dehydrogenase, chloroplastic; CM, chorismate mutase; PAL, phenylalanine ammonia lyase; C4H, cinnamate 4-hydroxylase; 4CL, 4-coumarate-CoA ligase; HCT, hydroxycinnamoyl shikimate transferase; C3H, p-coumarate 3-hydroxylase; CCOAMT, caffeoyl-CoA 3-O-methyltransferase; CCR, cinnamoyl CoA oxidoreductases; COMT, caffeic acid 3-O-methyltransferase; CHS, chalcone synthase; CHI, chalcone isomerase. ① Showing the ferulic acid biosynthesis via CCOAMT sub-pathway; ② Showing the ferulic acid biosynthesis via COMT sub-pathway;③ Showing the flavonoid biosynthetic sub-pathway (Dong et al., 2022; Li et al., 2023).

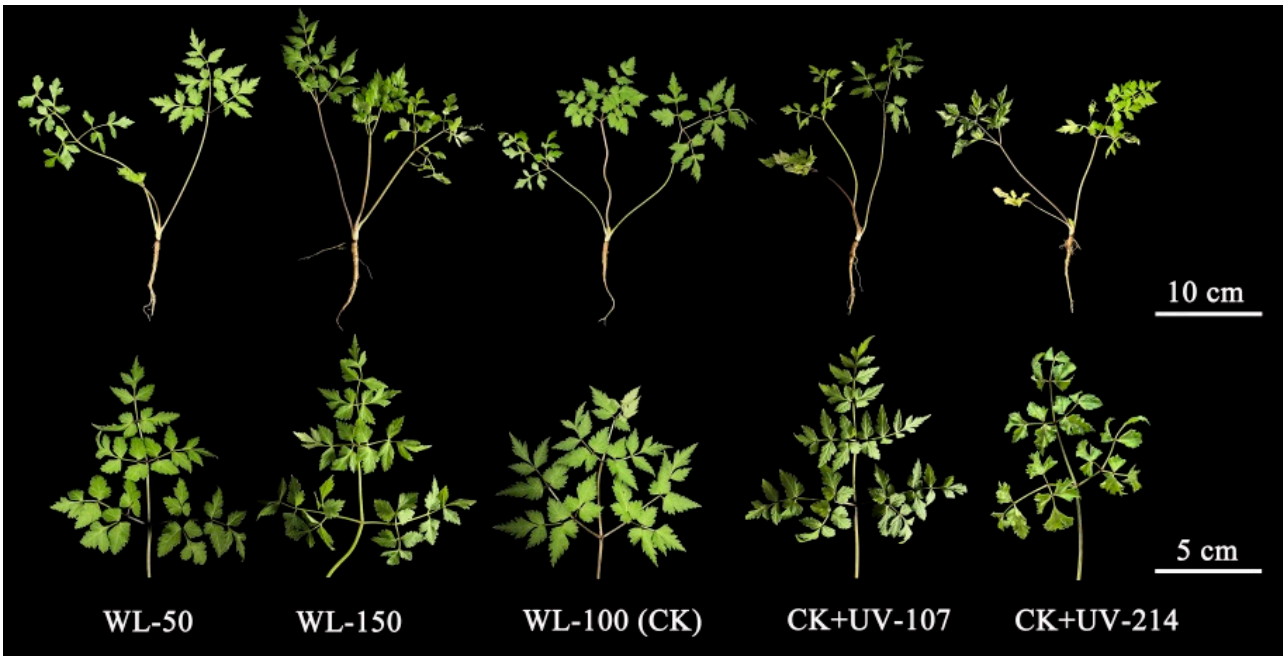

Figure S2. Morphological characteristics of *A. sinensis* in response to WL and UV-B.

Table S1. Stomata parameters of *A. sinensis* in response to WL and UV-B (mean ± SD, n=10).

Stomata parameters	WL-50	WL-150	WL-100	CK+UV-107	CK+UV-214
Density (mm^2)	10.31 ± 0.71[c]	14.45 ± 0.76[a]	12.75 ± 0.76[b]	9.21 ± 0.84[d]	8.14 ± 0.67[e]
Opening percentage (%)	19.10 ± 0.06[cd]	42.42 ± 0.12[a]	37.68 ± 0.12[ab]	27.73 ± 0.06[bc]	16.25 ± 0.08[d]

Note: Different letters showing a significant difference at $p < 0.05$ level among different treatments.

Table S2. Primer sequences used to amplify the 49 genes related to the biosynthesis of volatile oils, ferulic acid, flavonoids, and polysaccharides.

Gene name	Primer sequences (5' to 3')	Amplicon size (bp)
ACT	Forward: TGGTATTGTGCTGGATTCTGGT	109
	Reverse: TGAGATCACCACCAGCAAGG	
**Volatile oils biosynthesis (19)**		
LOX3.1	Forward: TGCTGATGGCGTTATCGAGT	138
	Reverse: TCTGGTACTGCCATTCCCCT	
NCED1	Forward: TGCCACCGTCTTCCAGTTAC	103
	Reverse: AGTGATGTCCTGCAGTTGGT	
ADH1	Forward: AGGTTGACCCGCAAATTCCT	148
	Reverse: GCAACCGCTAACCCAATCAC	
OPR3	Forward: CACTACCGTCAGGCTGCAAT	131
	Reverse: CCACCATACTCGTCTGTGCG	
CYP71D95	Forward: TGTCGGCCCTGATGAAACAT	155
	Reverse: TGTGTGCAAACGAAGGGTTTC	
CYP85A1	Forward: GATGTCATTCATGCGCTGCT	175
	Reverse: CAGGGTTGTCGCTGAGGTAT	
CYP86B1	Forward: GGCATCTCAACCTGGGAAGT	187
	Reverse: TCGGAAAATGGCTGTCCTCT	
CYP705A1	Forward: GAAGCGAACTGCGAACAACA	161
	Reverse: GCAACCCTACCACCCCAATC	
CYP94C1	Forward: TGTTTGGCCAGATGGTACGG	121
	Reverse: CCACCTCTCCGGCCTATACT	
BAMT	Forward: AAAAGCGAGTCCTAGCAGTGT	157
	Reverse: ATCTTCGCTGGTGGGATCTG	
ASAT3	Forward: AGCTGCTTCACATTCCGGTT	142
	Reverse: TTTCGCCTCACTGTCGTTGT	
BEAT	Forward: AAGCCGGCTCAAGTACCATC	439
	Reverse: AGAACATTTCCTCGGGGCTC	
TPS4	Forward: AACTTGAGGATGGCTCGTGG	120
	Reverse: TTTGTTCTTCGCCAACGTCC	
AIMT1	Forward: CTGGACTGACGAAGAAAGCGT	142
	Reverse: ATCCACCTGCGATGTGTTCT	
FPS1	Forward: GCTTGTGCGCTTCTTATGGC	120
	Reverse: TGCACCAAAGCAATCCAGGT	
CHLP	Forward: CCACGCCCGAAGAGACTATC	117
	Reverse: CGCACACATTCTCCCACTCT	
ZFPS	Forward: ACAACAAAGGCCAACACTGG	116
	Reverse: TGAAGATGTCCGCTCATGGT	
PMK	Forward: ACCGGAAGCAGATTGAAGCC	142
	Reverse: CCCAGTCCAGTTTTTGCCAC	
DXS	Forward: CAGAGCTGGGCTAGTTGGAG	198
	Reverse: TCTACGCCAATCCCGTTTCC	
**Ferulic acid biosynthesis (6)**		
PAL2	Forward: TCAACGGTGAACTCCATCCC	117
	Reverse: GCATCAATGGGTAGGTTGCG	
CYP73A10	Forward: CAATCGGACGTTTGGTGCAG	129
	Reverse: TTGGCTTGCAAACGATGGTG	
4CLL6	Forward: ACTCACTTCCCGGTTGTACC	107
	Reverse: GCAGCACCACATGAAACCTG	
HCT4	Forward: TTGCACTAGGATGTGGGGTG	158
	Reverse: CACTGGAGTAGGTGGATCGC	
COMT	Forward: TTATCCTGGTGTGGAGCACG	104
	Reverse: AATGTGCATCGCTCCAATCG	
CCOMT	Forward: GAGCCAGCTGATGCTCCTATG	132
	Reverse: CAGGGTAACTCCATCACCCAC	
**Flavonoid biosynthesis (18)**		
CHS2	Forward: CCGGATCTATCCCTTGAGCG	110
	Reverse: GTAAGGCCCACTTCACGGAG	

CHI2	Forward: CAAATTTTCCTCCGGGCTCC<br>Reverse: AGTCCAGTACTGCTTCTGCC	144
F3H-3	Forward: GCGTCCTCGAGTCTCTCAAC<br>Reverse: CCTCTCCCGTTCCTCGAATG	143
CYP75A5	Forward: TGATAGCGAAGGTGGGAAGC<br>Reverse: CCTGGGCTAAGACTTTCGGG	143
CYP71A12	Forward: GCAAGACCTTGTGGTTGCTG<br>Reverse: TTTCGGTGAGGAAGCAGTCC	162
CYP97B3	Forward: GGCTCGGTGTACAAACTTGC<br>Reverse: TTCGCTGTTTCCATGTGCTG	184
DFRA	Forward: TGAGATTCGTCGTGGTGTCG<br>Reverse: AGTAGTAAGCGAGCAAGCCC	160
UGT85C1	Forward: AGCATCTCAACTGCATTGGC<br>Reverse: GCAAGTCCCCAACCAAACTC	157
FLS1	Forward: ACCACCGAACTACCGTGAAC<br>Reverse: ATGAGCTTTGAGAGGGGTCC	100
GT6	Forward: TTTACGACTGGTGGAGCGAC<br>Reverse: TCCGCTGAGTTCCAATGCTT	168
ANS	Forward: ACAGCACTATCACCGCTCAC<br>Reverse: ATGTATCTTCCCTGCGCTGT	134
LDOX	Forward: ACGAGCTAAGAGGGCTAACG<br>Reverse: GCTTCAACACCAAGAGCCAG	172
RT	Forward: TTTGGAGCGGAGACGAAACT<br>Reverse: AAACAATCCCACGCCCTCTC	175
RhGT1	Forward: AGCAATTTCCGATGGGCTCT<br>Reverse: TGAGCCATACCAAGCACTCG	116
3MAT	Forward: CAGGCCCTCAAGAGCACAAT<br>Reverse: AATCGTAGCCCGCGTTTTTG	114
3AT1	Forward: ACCGCAAGCGCAAATGTTAG<br>Reverse: CACTCTCCATTGCCCCTGTT	139
F3GT1	Forward: GCTTTGGAACTGTGGCGATG<br>Reverse: AGGCCACGATTTTTCCGGTT	165
CYP76AD1	Forward: TCCCTTCTTAATACCGCGCC<br>Reverse: TCGCGTCCTATGATCCATGC	105
**Polysaccharides biosynthesis (6)**		
AGAL2	Forward: GTATCAACCGGACTTGCTGC<br>Reverse: GAAATGTTGAGCGTCTGGGC	112
GOLS1	Forward: TACGGCAATGTGGACGAACT<br>Reverse: GGGCATTGTTGGCAGTAACC	119
INVA	Forward: TGTTGAGGGAAGCGGTTGTT<br>Reverse: GCAGACGGAACAAAATCCCG	124
SUS2	Forward: TGTGGAAGCCATGACCTGTG<br>Reverse: GCCATGAGATCAGCAGCCTT	132
TPS5	Forward: AGCGAGGTTACATTGGCCTT<br>Reverse: TCAACCCCAAGCAACACAGT	172
UXS5	Forward: CTGTGGAGAACACACCCGAT<br>Reverse: TCGTTTTCCATGAGGGGAAGG	121
